# Radiation, inflammation and the immune response in cancer

**DOI:** 10.1007/s00335-018-9777-0

**Published:** 2018-09-03

**Authors:** Kelly J. McKelvey, Amanda L. Hudson, Michael Back, Tom Eade, Connie I. Diakos

**Affiliations:** 1 Bill Walsh Translational Cancer Research Laboratory, Northern Sydney Local Health District Research and the Northern Clinical School, University of Sydney, St Leonards, NSW 2065 Australia; 2 Sydney Neuro-Oncology Group, North Shore Private Hospital, St Leonards, NSW 2065 Australia; 3 Sydney Vital Translational Research Centre, Royal North Shore Hospital, St Leonards, NSW 2065 Australia; 4 Northern Sydney Cancer Centre, Royal North Shore Hospital, St Leonards, NSW 2065 Australia

## Abstract

Radiation is an important component of cancer treatment with more than half of all patients receive radiotherapy during their cancer experience. While the impact of radiation on *tumour morphology* is routinely examined in the pre-clinical and clinical setting, the impact of radiation on the *tumour microenvironment* and more specifically the inflammatory/immune response is less well characterised. Inflammation is a key contributor to short- and long-term cancer eradication, with significant tumour and normal tissue consequences. Therefore, the role of radiation in modulating the inflammatory response is highly topical given the current wave of targeted and immuno-therapeutic treatments for cancer. This review provides a general overview of how radiation modulates the inflammatory and immune response—(i) how radiation induces the inflammatory/immune system, (ii) the cellular changes that take place, (iii) how radiation dose delivery affects the immune response, and (iv) a discussion on research directions to improve patient survival, reduce side effects, improve quality of life, and reduce financial costs in the immediate future. Harnessing the benefits of radiation on the immune response will enhance its maximal therapeutic benefit and reduce radiation-induced toxicity.

## Introduction

The use of ionising radiation (IR) in the treatment of cancer has existed since the early 1900s, since the realisation that the disposition of energy from photons, X-rays or gamma rays into cells and tissue leads to the death of cancer cells. Since then, radiation’s inclusion in treatment paradigms has seen dramatic improvements in cancer survival. Radiation therapy (RT) outcomes in the last 20 years have improved dramatically with improved targeting by image guidance (Jaffray 2012), target volume delineation through positron-emission-tomography and advanced magnetic resonance imaging (McKay et al. 2018) and more precise treatment delivery to these targets through computerised 3D planning and beam modulation (Nutting et al. 2011). This has allowed radiation doses to be increased, tumour control improved, and side effects dramatically reduced. Despite improvements in outcomes for most cancers, biomarkers that assist in selecting patients in whom radiation will be successful, and is associated with quality of life and not treatment-limiting side effects, remains elusive. Changes here will be dependent upon understanding the cellular and molecular response of the tumour microenvironment to radiation.

The importance of the role of inflammation in patients with malignancy was epitomised by the inclusion of inflammation in the revised “Hallmarks of Cancer” (Hanahan and Weinberg 2011). In the clinical and research setting, a comprehensive understanding of IR and its ability to induce and modulate inflammation and the immune system remains largely in its infancy, but in order to improve patient survival, a better understanding is essential. In doing so, we may be able to better select patients who will benefit from RT, select the optimal RT dose and fractionation regimen, or be able to augment the response by altering the microenvironment with emerging targeted therapies and/or immunotherapies (Lan et al. 2018; Zhang and Niedermann 2018).

Here, we discuss how IR initiates and influences the inflammatory/immune system in the tumour microenvironment, and modulates immune cell populations. The critical role RT plays in the re-activation of the immune response for immediate and long-term cancer eradication will be discussed, with its role as a key adjuvant to upcoming targeted and immunotherapies, where a greater understanding is required if we are to improve global cancer survivorship.

## Radiation-induced immune mediators

**Fig. 1:**
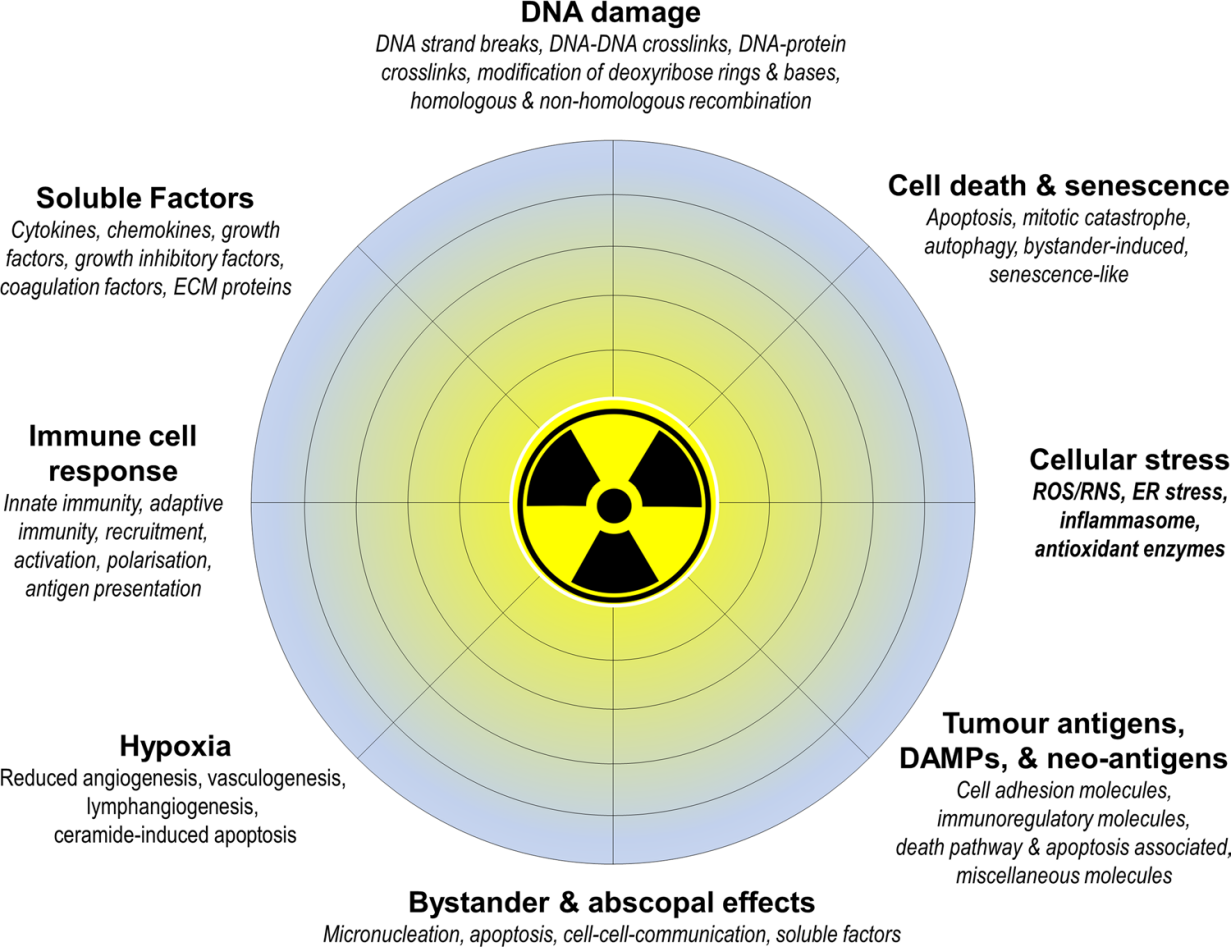
Radiation-induced factors that initiate and modulate the inflammatory/immune response

The current state of knowledge on the radiation-induced biological factors that can initiate a pro-inflammatory immune response within the tumour microenvironment are presented in (Fig. 1).

### DNA damage, reactive oxygen/nitrogen species, ER stress and hypoxia

#### DNA damage

The old adage that radiation inflicts DNA damage primarily through direct interaction with macromolecules (nucleic acids, lipids, proteins) has long been dismissed. Only an estimated one-third of DNA damage is caused by the direct interaction of X-ray and γ-ray radiation hitting the macromolecule; the remaining two-thirds are due to indirect effects mediated by reactive oxygen/nitrogen species (ROS/RNS) generation (Kang et al. 2012). DNA damage includes DNA strand breaks, DNA–DNA crosslinks, DNA–protein crosslinks and modification of the deoxyribose rings and bases. Estimates of the number of DNA double-strand breaks (DSB) in mammalian normal diploid cells per 1 Gy of IR range from 25 to 40 (Lobrich et al. 1994a, b; Olive 1999) to 1815 per cell (Buatti et al. 1992). This number varies greatly depending on the radiation type due to differences in the linear energy transfer (LET) of the irradiating photon/particle, a measure of the amount of energy the particle deposits as it traverses a unit of distance, and its subsequent relative biological effectiveness (RBE; Table 1). X-ray and γ-ray are sparsely ionising with low LET/RBE. They induce fewer single and DSB, and enable greater DNA repair whether it be homologous or non-homologous (Mitteer et al. 2015). In line with this, X-ray and γ-radiation requires high doses to elicit cell death. In contrast, particle and heavy ion radiation (emitting α and β particles) are densely ionising with high LET/RBE inducing markedly more DSB for the same radiation dose (Table 1). Where the DSB exceed the cell’s capacity for DNA repair cell death mechanisms are activated (see “Cell death and senescence”).

**Table 1:** Historical and current IR types used for cancer RT

	Source^a^	Average energy	RBE
External beam therapy			
γ-ray	^60^Co, ^137^Cs, ^192^Ir	0.4–1.3 MeV	1.0
X-ray	W	4–18 MeV	1.0
Particle and heavy ion therapy			
Proton	H-ion	70–250 MeV	10–32
Neutron	^10^B	50–70 MeV	10–32
Heavy ion	C-ion	450 MeV	20
Radionuclide therapy			
α-particle	^4^He, ^213^Bi, ^223^Ra, ^225^Ac, ^238^Pu	5.5 MeV	10–20
β-particle	^106^Ru, ^131^I, ^177^Lu	10–200 keV	1.0-1.7

*Ac* actinium, *B* boron, *Bi* bismuth, *C* carbon, *Co* cobalt, *Cs* caesium, *H* helium, *I* iodine, *Ir* iridium, *keV* kilovolt, *Lu* lutetium, *MV* megavolt, *MeV* megaelectronvolt, *Pu* plutonium, *Ra* radium, *Ru* ruthenium, *RBE* relative biological effectiveness, *W* tungsten

^a^Superscript numbers denote the atomic mass (number of protons and neutrons) of the element

As an example, irradiation of human peripheral blood lymphocytes induces threefold more cytotoxicity with proton radiation (dose rate [DR] 4.5 Gy/min), compared to X-ray irradiation (DR 9 Gy/min) across a dose range of 0.3–4 Gy (Miszczyk et al. 2018). Similar results were obtained with patient-derived glioma stem cells, where doses of 0–10 Gy proton irradiation (DR 2 Gy/min) induced more single and DSB, and ~ 25% greater apoptosis when compared to X-ray radiation (DR 2 Gy/min). Akin to photon irradiation, induction of apoptosis by proton therapy is partially mediated by a sevenfold up-regulation of ROS species 3 days after irradiation (Mitteer et al. 2015). These results are based on single dose fractionation (1fx). The RBE of radiation depends on the LET, cumulative dose, number and timings of fractions and the radiosensitivity of the targeted cells or tissues.

External beam RT, delivered in fractionated regimens, induces an accumulation of DNA strand breaks due to the failure to repair all breaks after each fraction, and is similar in acute- and late-responding tissues (Rube et al. 2010). Mice X-ray irradiated with 6 Gy/3fx or 10 Gy/5fx showed 3–4-fold more DSB compared to 2 Gy/1fx (DR 2 Gy/min) (Rube et al. 2010). Human fibroblast cells irradiated with 6 Gy/4fx show greater cytotoxicity than a single dose of 6 Gy (Rezacova et al. 2011). In contrast, irradiation of lymphocytic leukaemic cells with 4 Gy/4fx, compared to 4 Gy/1fx, showed similar survival. This may indicate that a minimum of 1.5 Gy per fraction is required to elicit a biological response, and those cells with a high proliferative index are less responsive to ‘conventional’ fractionated treatment due to an ability to undergo DNA repair. Hypo-, hyper-fractionation and ‘ultra’-fractionation RT has shown some pre-clinical and clinical benefit over convention fractionation regimens in its modulation of the inflammatory response, in addition to alterations in DNA repair (Barlow et al. 2016; Stuschke and Thames 1997; Whelan et al. 2010).

#### ROS/RNS

IR passing through living tissues generates reactive oxygen/nitrogen species (ROS/RNS) and highly reactive hydroxyl (OH^·^) and nitrosyl (NO^·^) radicals, superoxide (O_2_^·−^), peroxynitrite (ONOO^−^), and hydrogen peroxide (H_2_O_2_). Each of these can damage cellular macromolecules including DNA, proteins or membranes via single and DSB, modification to deoxyribose rings and bases, intra- and inter-strand DNA–DNA crosslinks, and DNA–protein crosslinks. These changes are deleterious if the cell is unable to repair the damage, or misrepairs the damage. The high cytotoxic efficacy of IR is attributed largely to ROS generated within 2 nm of DNA and the formation of complex DSBs. In glioma stem cells, a 50% (X-ray) and 200% (proton) increase in ROS/RNS generation underpinned DNA damage and cell cycle changes leading to radiation-induced apoptosis (Mitteer et al. 2015). Proton and X-ray irradiation-derived ROS induced single and DSB, phosphorylation of histone 2AX and checkpoint kinase-2, reduced cell cycle recovery from G2 arrest, and activated caspase-3 and poly(ADP-ribose) polymerase cleavage leading to cell death (Mitteer et al. 2015).

**Fig. 2:**
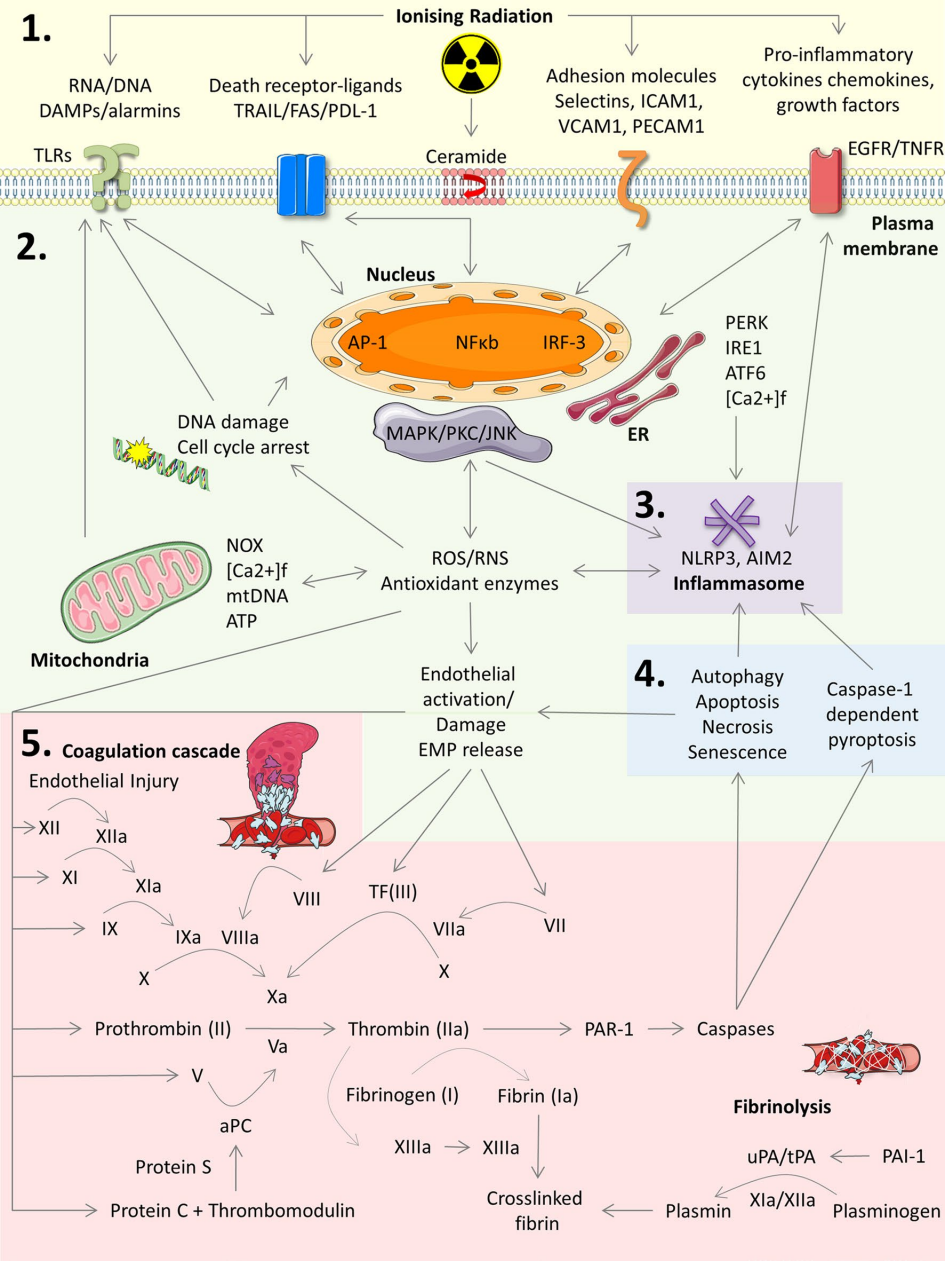
Schematic overview of the interconnected network of inflammatory and immune response pathways activated by IR. Zones represent the topics discussed in this review; (1) cytokines, growth factors, adhesion molecules (“Cytokines, chemokines, growth factors, adhesion molecules and coagulation factors” section), (2) DNA damage, ER stress, ROS/RNS, hypoxia (“DNA damage, reactive oxygen/nitrogen species, ER stress, and hypoxia” section), (3) inflammasomes (“Inflammasomes”), (4) cell death and senescence (“Cell death and senescence” section), and (5) coagulation and fibrinolysis (“Cytokines, chemokines, growth factors, adhesion molecules and coagulation factors” section). Straight arrows denote activation. Two-headed arrows denote bidirectional activation. Curved arrows denote a catalytic or enzymatic action. Figure prepared using Servier Medical Art (https://smart.servier.com/)

Radiation-induced ROS also modulate cellular changes through non-nuclear interactions summarised in (Fig. 2) by (i) damaging mitochondria, activating the nicotinamide adenine dinucleotide phosphate oxidase-1/Rac-1 pathway(Kang et al. 2012; Narayanan et al. 1997; Tateishi et al. 2008) or other oxidases; (ii) release of damage-associated molecular pattern proteins (DAMPs) and purinergic signalling molecules adenosine and adenosine triphosphate (ATP)-mediated by the purinergic receptor 2 × 7 (Ohshima et al. 2010); (iii) alterations in nuclear and cytosolic calcium flux ([Ca^2+^]_f_) homeostasis responsible for cell cycle progression (Todd and Mikkelsen 1994); (iv) activation of ROS responsive protein kinases such as protein kinase c (PKC), mitogen-activated protein kinase (MAPK), apoptosis signal-regulating kinase-1, and the thioredoxin redox-sensing system (Fujino et al. 2006); (v) activation of ‘cytokine-induced cell death’ mediated by members of the TNF superfamily, including TNF-α/β, Fas, TNF-related apoptosis-inducing ligand (TRAIL), TNF-like weak inducer of apoptosis (TWEAK), and TNFSF15 (Shakibaei et al. 2005); (vi) changes in expression of adhesion molecule, cytokine, chemokines, growth factor, proteases; (vii) activation of inflammasomes (see “Inflammasomes” section); and (viii) activation of the nuclear factor erythroid-2-related factor-2 oxidative stress and antioxidant pathways (Bravard et al. 1999; McDonald et al. 2010; Tsukimoto et al. 2010).

The effects of ROS are amplified by the interaction of ROS/RNS with lipids, membranes and oxygen, and convergence with cytokine, DAMP and endoplasmic reticulum (ER) stress pathways (Fig. 2). In concert, these create a cyclic and self-amplifying cascade which contributes to a pro-inflammatory tumour microenvironment, bystander effects (see “Bystander and abscopal effect” section), and radiation-induced adverse events.

#### ER stress

Aside from nuclear interactions, IR can induce cytosolic effects altering the normal functioning of cellular ER, mitochondria and inflammasomes.

Dysfunction of ER protein folding activates protein kinase RNA-like ER kinase (PERK), activating transcription factor 6 (ATF6), and inositol-requiring enzyme 1 (IRE1) cell stress and unfolded protein response (UPR) pathways. These pathways aim to alleviate cellular injury but if exceeded trigger cell death mechanisms. 15 Gy ^137^Cs γ-irradiation of human umbilical vein and coronary artery endothelial cells (DR 3.51 Gy/min) activated the PERK/eukaryotic initiation factor 2α/ATF4 UPR signalling pathway (Kim et al. 2014). ^137^Cs γ-radiation (DR 0.79 Gy/min) of HL-7702 hepatoma cells activated the binding immunoglobulin protein (BiP)/PERK/eukaryotic initiation factor 2α ER stress signalling pathway (Xie et al. 2016). X-ray radiation (DR 2.5 Gy/min) of human D54 and LN827 glioblastoma cells activated the ATF6/BiP/NOTCH1 ER stress pathway mediated by 45–78% elevations in ROS levels (Dadey et al. 2016). 2 Gy carbon ion radiation of in vitro S180 cells and in vivo S180 sarcoma-tumour-bearing mice induced CHOP and BiP expression, and activation of the IRE1/c-Jun N-terminal kinase (JNK)/B-cell lymphoma (Bcl)-2/beclin-1/p62 ER stress pathway leading to autophagosome formation and apoptosis (Zheng et al. 2017). This effect was also achieved with 4 Gy X-ray irradiation. These data show that cell and/or radiation type may determine the ER stress pathway leading to the triggering of cell death mechanisms (see “Cell death and senescence” section). Interestingly, both the PERK/eIF2a and IRE1/JNK ER stress pathways can trigger apoptosis and autophagy (Moretti et al. 2007).

In addition to ER stress and UPR pathways, irradiation of cells and tissues alters mitochondrial and inflammasome function. 4 Gy ^137^Cs γ-radiation of rat brains induced DNA strand breaks, and mitochondrial cytochrome c and second mitochondria-derived activator of caspases release which activated caspases-3, -8 and -9 (Li et al. 2014). 3 Gy ^137^Cs γ-irradiation (DR 0.83 Gy/min) of human HepG2 hepatocyte cells induced p53-dependent cytochrome c, NO and ROS release (He et al. 2011, 2012). The nucleotide-binding domain and leucine-rich repeat (NLRP3) and absent in melanoma (AIM)-2 inflammasomes form an integral part of the pathway from radiation-induced DAMPs and ROS/RNS to activated cytokines and are discussed in “Inflammasomes” section.

#### Hypoxia

Regions of hypoxic tissue are common in solid tumours and are thought to contribute to radiation resistance. Irradiation leads to a 20% loss of endothelial cells at 24 h (Pena et al. 2000) and a 46.5% reduction in tumour perfusion at 3-day post-radiation (El Kaffas et al. 2018). However, fractionated RT regimens can lead to re-perfusion, decreasing hypoxia within tumour supporting the 6 R’s of fractionation; repair, repopulation, reoxygenation, redistribution, radiosensitivity and remote bystander effects. Endothelial cells contain very high level of ceramide synthase and acid-sphingomyelinase (Marathe et al. 1998), which following high-dose irradiation (8–10 Gy) lead to large amounts of acid-sphingomyelinase-induced ceramide production and microvascular endothelial cell apoptosis (Paris et al. 2001; Pena et al. 2000). Lower doses of radiation, 0.5 Gy ^137^Cs γ-radiation, also result in ceramide-induced apoptosis in bovine aortic endothelial cells (Haimovitz-Friedman et al. 1994). Loss of the blood microvasculature and oxygenation leads to the formation of hypoxic regions which can recruit immune cells and increase ‘bystander’ cytotoxicity. Murine prostate TRAMP-C1 tumours irradiated with 25 Gy/1fx or 60 Gy/15fx showed a progressive decrease in microvascular density associated with decreases in CD31, endoglin, and tyrosine kinase with immunoglobulin-like and EGF-like domains 1 gene expression. Hypoxic regions surrounded centrally located dilated vessels and demonstrated infiltration and aggregation of CD68^+^ tumour-associated Mɸ (Chen et al. 2009). Tumour hypoxia enhances ROS-mediated cytotoxicity. ROS in conditioned media from irradiated HepG2 hepatocyte cells grown under hypoxic conditions showed enhanced micronuclei formation and apoptosis in non-irradiated HL-7702 hepatoma cells, compared to normoxic conditions (Xie et al. 2016).

Endothelial cells of the lymphatic vasculature are also injured by IR. 0, 15, or 30 Gy lead to the loss of lymphatic capillaries and a dose-dependent increase in lymphatic endothelial cell apoptosis (Avraham et al. 2010). Hypoxia increases CXCL12/CXCR4-mediated adherence of non-small cell lung carcinoma cells to lymphatic endothelial cells (Irigoyen et al. 2007), which may serve as a means of tumour metastases. While surgery is a primary contributor to the development of lymphedema in cancer patients, RT is also an independent risk factor with a tenfold increased risk in some patients (Hinrichs et al. 2004; Petrek et al. 2001). These data show that hypoxia can contribute to increased tumour cell death, but has also been implicated in tumour radio-resistance, tumour metastases and lymphedema as a long-term radiation-induced adverse event.

### Cell death and senescence

The sensitivity of cells to radiation-induced cell death (loss of clonogenic capacity), as well as the type and timing/rate of cell death are determined by DNA damage repair/response system and the activation of specific groups of genes which control cell cycle checkpoints, inhibition of replication and transcription, induction of apoptosis, or an adaptive immune response (Szumiel 1998; Watters 1999). Probably, the most widely reported DNA damage sensors are tumour suppressor p53, poly(ADP-ribose) polymerase, DNA-dependent protein kinase, ataxia telangiectasia mutated protein, and to a lesser extent BRAC1/2, interferon regulatory factor-1, retinoblastoma, and cyclin kinase inhibitor p21^WAFI/CIP1^ (Szumiel 1998; Watters 1999). Irradiation of cancers induces a continuum of non-immunogenic/tolerogenic and immunogenic cell death mechanisms; autophagy, mitotic catastrophe, apoptosis, necrosis, ‘cytokine or bystander-induced’ and ‘senescence-like’ cell death which impacts the clinical outcome of RT (Lauber et al. 2012). However, there is still debate as to the immunogenicity of these different types of cell death (Gamrekelashvili et al. 2015). Irrespective of the mechanism of cell death, the generation of DNA damage, cell cycle arrest, cytokines, DAMPS, ROS/RNS, engagement of death receptors triggers, and activation of antigen presenting cells (see “Inflammatory and immune system cellular response” section) induces a cyclic pro-inflammatory tumour environment (Fig. 2).

It is interesting to note that the mechanism of cell death is influenced by radiation type as much as the above biological factors (Miszczyk et al. 2018). This is likely in part due to the difference in LET/RBE (Table 1) and therein the number of DNA strand breaks between the two radiation types. Wider studies and comparisons on how tumour and healthy cells die following irradiation of different radiation types, fractionation, and spatial modulation alone and in combination with various adjuvants—chemotherapy, targeted therapy, immunotherapy, oncolytic viruses, and vaccines are required.

#### Autophagy

Autophagy is a cytoprotective and cytocidal mechanism, whereby a cell digests a portion of cytoplasmic components to sustain cell metabolism and overcome radiation damage to macromolecules and organelles (Rubinsztein et al. 2007). Autophagy is regulated by a set of evolutionarily conserved genes which control autophagy induction, cargo packaging, vesicle nucleation, and expansion, retrieval, docking and fusion, and finally vesicle breakdown (Levine and Klionsky 2004). Inactivation of the autophagy and tumour suppressor gene beclin 1 induces spontaneous tumour formation in mice (Qu et al. 2003; Yue et al. 2003) and is monoallelically deleted in 40–75% of breast, ovarian and prostate cancers (Aita et al. 1999).

Radiation induces ROS-dependent and -independent DNA, lipid and protein damage in cells, as well as activating the ER stress pathway; both are capable of inducing autophagy (Chaurasia et al. 2016). 2–20 Gy ^137^Cs γ-radiation (DR 3.8 Gy/min) of malignant human glioma cell lines showed temporal and dose-dependent response patterns of autophagy and apoptosis from day 1 to 5 post-irradiation (Jo et al. 2015). Radiation induced autophagy at days 1–7 post-irradiation for 5–20 Gy, and apoptosis from day 3 in lethal doses (10 and 20 Gy) and day 7 in sub-lethal doses (2 and 5 Gy). These data show that there are temporal changes in the cell death mechanism with an attempt at non-immunogenic cell survival/cytoprotection (autophagy) made prior to the initiation of apoptosis, particularly with fractionated doses (< 5Gy). This may limit the initial inflammatory/immune response due to the relative absence of immunogenic tumour antigen, and supports tumour progression and recurrence.

#### Mitotic catastrophe

Mitotic catastrophe is delayed or aberrant mitosis followed by apoptosis, necrosis or complete fragmentation of interphase nuclei with eventual cell death. Morphologically, mitotic catastrophe is associated with the formation of multinucleate giant cells with uncondensed chromosomes. Irradiation leads to a range of disruptions to the cell cycle. For ~ 6 h following 0.5–4 Gy X-ray irradiation, the activated G_1_–S checkpoint fails to efficiently prevent S-phase entry by cells with unrepaired DSBs and other DNA damage. At > 6 h, the checkpoint is inefficiently maintained leading to the formation of chromosome breaks at doses of 1–4 Gy during the G_2_–M phase(Deckbar et al. 2010). Similar effects to the p53-dependent G_1_–S checkpoint can be found with 5 Gy γ-radiation using mouse embryonic fibroblast cultures (Cann and Hicks 2006). When these cells enter M-phase, aberrant mitosis directly induces cell death via caspase-2 activation, and/or indirectly through the release of mitochondrial cytochrome c and apoptosis-inducing factor, and subsequent activation of caspase-9 and -3.

Cells show a significant difference in the susceptibility of DNA strand breaks to radiation, and in the ability to repair these breaks during different phases of the cell cycle. This concept underpins the use of fractionated RT in clinical practice. Using 3.5 Gy ^137^Cs γ-radiation, McArt and colleagues show that cervical cancer cells (HeLa) were most susceptible to DNA damage during S-phase, and least during M-phase. Conversely, repair was most efficient during G1-phase and least during M phase (McArt et al. 2010). The level of DSB induced by X-ray radiation during G_1_ or M phase does not correlate with cell killing (Iliakis and Okayasu 1988) with some cells of incomplete mitosis undergoing post-mitotic apoptosis; single fraction γ-radiation induced apoptosis in non-clonogenic cells three cell cycles after irradiation of normal human fibroblast and epithelial cells (Linke et al. 1997).

#### Apoptosis

This traditional ‘programmed cell death’ is induced by radiation via intrinsic mechanisms—DNA damage sensors, cytokines, stress response, DAMPs, ceramide and ROS/RNS initiated signalling pathways discussed elsewhere, or extrinsic mechanisms—up-regulation of death receptors (Krysko et al. 2012). Radiation-induced apoptosis can be mediated by the up-regulation and engagement of death receptors Fas ligand (FasL), TRAIL, and programmed death-ligand-1 (PD-L1) or their corresponding receptors. 2.5–10 Gy γ-radiation induced dose-dependent apoptosis, increased expression of TRAIL, TRAIL-R2, Fas, p53, and down-regulation of anti-apoptotic protein Survivin in neuroblastoma stem cells 6 h after RT (Ivanov and Hei 2014b). Radiation doses of 8–20 Gy up-regulate Fas on tumour cells (Chakraborty et al. 2003; Garnett et al. 2004) to mediate CD8^+^ cytotoxic T-cell (CTL) killing. 2–5 Gy γ-radiation induced low levels of apoptosis and induced a strong ‘bystander effect’ in non-irradiated cancer cells (Ivanov and Hei 2014b).

Investigation of 10 and 20 Gy/1fx irradiation dose-dependently up-regulated surface expression of Fas on 10 out of 23 different human carcinoma cell lines (6/12 colon, 2/7 lung, and 2/4 prostate) after 72 h (Garnett et al. 2004). In another study, repeat biopsies of lymphoma and squamous cell carcinomas after 4, 10, and 20 Gy cumulative dose RT showed absent to very low expression of Fas on the 52 squamous cell carcinomas, whereas 2/4 malignant lymphoma showed high Fas expression after 4 and 10 Gy RT (Ogawa et al. 1997). γ-irradiation induced up-regulation of Fas and induction of G_1_ cell cycle arrest is dependent on p53 activity (Sheard et al. 1997). Combined, these studies indicate that radiosensitivity of cancers is type specific and may relate to Fas expression, whereby Fas/FasL expression can be up-regulated in some cancers by high-dose single fraction or low-dose fractionated RT (Ogawa et al. 1997; Sheard et al. 1997). Identification of cancers types/subtypes or patients that do not up-regulate TRAIL/Fas expression following RT may help direct radiation treatment selection.

#### Necrosis

Of all the cell death mechanisms, necrosis is the most morphologically distinct, with profound cellular damage including clumping and degradation of DNA, swelling and rupture of the plasma membrane, organelle degradation, mitochondrial swelling, increased vacuolation and the release of DAMPs (Gamrekelashvili et al. 2015). Necrotic cell death was observed at doses of 0.3–4 Gy from both proton and X-ray radiation (Miszczyk et al. 2018). The radiation type influenced the ratio of apoptotic:necrotic cell death with 25:75% observed for proton therapy, and 40:60% observed for X-ray therapy at 4-h post-irradiation. This suggests a greater tendency for necrosis with proton therapy. 5 Gy irradiation of human MG-63 osteosarcoma spheroids died by apoptosis through triggering Bcl-2, Bax and Bcl-xL in the apoptotic signalling pathway, while higher doses of 30 Gy induced necrotic cell death (Rainaldi et al. 2003). Combining these data shows that radiation dose and type influence the mechanism of cell death in tumour cells, likely attributable to the number of DSBs. This has implications for the selection of patients for appropriate RT regimens. For example, as well as the reduced entry/exit dose of radiation, ‘cold/low-immunogenic’ tumours such as glioblastoma may benefit more from proton therapy than conventional external beam RT, as apoptosis is considered less immunogenic than necrosis.

#### ‘Cytokine’-induced, ‘Bystander’-induced, and ‘Senescence-like’ cell death

Cytokine engagement of receptors on neighbouring cells can trigger a cytokine cascade that upregulates death receptors on the cell surface, and/or induces cell death; IL-1α, IL-6, IL-8, TGF-β1, TNF-α/β (Burdak-Rothkamm et al. 2007; Dong et al. 2015; Ivanov and Hei 2014a; Narayanan et al. 1999; Shao et al. 2007, 2008; Shareef et al. 2007). The interconnected network of pro-inflammatory cytokines, ROS and apoptotic ligands contributes to ‘cytokine’- and ‘bystander’-induced cell death. Bystander-induced cell death includes a wider range of initiating molecules secreted by irradiated neighbouring cells. These include the cytokines listed above and ROS/RNS responses NO^·^, O_2_^·−^, H_2_O_2_ (Burdak-Rothkamm et al. 2007; He et al. 2012; Narayanan et al. 1997; Shao et al. 2007, 2008, 2003); ER stress response cytochrome c (He et al. 2011, 2012); and apoptotic pathways TRAIL, Fas, PDL-1, TWEAK and TNFSF15 (Ivanov and Hei 2014a, b; Lupu-Plesu et al. 2017; Shakibaei et al. 2005; Shareef et al. 2007).

As an alternative to cell death, cells with irreparable DNA damage are programmed to undergo premature senescence to maintain the integrity of the genome (so called senescent-like cell death) (Campisi and d’Adda di Fagagna 2007). Cells experience irreversible growth arrest, but the metabolic processes remain active. Such a response may prevent an immunogenic response to this ‘self-sterilising’ cell death. Senescent–like cell death is more common among cells of epithelial origin (Suzuki et al. 2001), compared to haematopoietic cells which predominantly undergo apoptosis due to the relative radiosensitivity of the cell types (Ross 1999). IR of normal human keratinocytes induced ROS-mediated premature senescence by the up-regulation of oxidase genes *Lpo, p22-phox, p47-phox* and *Gp91* (Dong et al. 2015). Normal human diploid cells irradiated with X-ray radiation lead to permanent cell growth arrest through the accumulation of p53 and induction of p21^WAFI/CIP1^ and p16. The senescent-like state was associated with expression of senescence-associated beta-galactosidase, but not telomere shortening (Suzuki et al. 2001). Four Gy radiation induced apoptosis in 65% of murine bone marrow cells (Meng et al. 2003). After 5 weeks, 33% of murine bone marrow cells survived irradiation but demonstrated a senescent phenotype; expression of senescence-associated beta-galactosidase, p53 and p21^WAFI/CIP1^, p16, and p19 and an inability to form colony forming units of granulocyte Mɸ (Meng et al. 2003). Endothelial cells can undergo senescence when exposed to IR. Irradiated endothelial cells decrease NO production and expression of thrombomodulin (TM), and elevate levels of ROS, inflammatory cytokines and adhesion molecules. Functionally, the cells show an inability to proliferate and form vascular structures (Wang et al. 2016).

### Cytokines, chemokines, growth factors, adhesion molecules and coagulation factors

Radiation activates the interconnected network of cytokines, adhesion molecule, ROS/RNS and DAMPs leading to a self-amplified cascade (Fig. 2), which generates pro-inflammatory, pro-oxidant tumour microenvironment and ultimately tumour cell death. Regulation of these molecules is controlled by transcription factors, tyrosine kinases and tumour suppressors. Table 2 summarises the inflammatory mediators within the tumour microenvironment that are modulated by IR.

**Table 2:** Tumour microenvironment inflammatory mediators modulated by IR

Mediators	Source	Dose/fraction	Tissue niche	References
Transcription factors				
NF-κB	α-particle (^241^Am)	1 Gy/1fx	Human bronchial epithelial cells (Beas-2B) conditioned media on macrophage cells (U937)	Fu et al. (2016)
NF-κB, AP-1, Sp-1, p53	γ-ray (^137^Cs)	5–30 Gy/1fx	Rat cerebral cortex	Raju et al. (2000)
NF-κB, AP-1, CREB	γ-ray (^137^Cs)	10 Gy/1fx	Rat brain	Lee et al. (2010)
STAT-3	γ-ray (^60^Co)	2–10 Gy/1fx	Human alveolar carcinoma cells (A549)	Gao et al. (2014)
NF-κB	α-particle (^3^He) microbeam	0.5 Gy/1fx	Human skin and lung fibroblasts	Zhou et al. (2008)
NF-κB	X-ray	2–8 Gy/1fx	Murine microglia cells (BV-2)	Hwang et al. (2006)
Tyrosine/protein kinases				
PKCβII	γ-ray (^137^Cs)	1 Gy/1fx or 10 Gy/1fx	Human lung fibroblast (MRC-5)	Baskar et al. (2008)
Raf-1, MAPK, PKCγ, PLC, IP_3_	γ-ray (^60^Co)	2 Gy/1fx	Human squamous carcinoma cells (A431)	Dent et al. (2003)
MAPK, JNK, ERK, p38	α-particle (^241^Am)	1 Gy/1fx	Human bronchial epithelial cells (Beas-2B) conditioned media on macrophage cells (U937)	Fu et al. (2016)
Tumour suppressor				
p53	γ-ray (^137^Cs)	5–30 Gy/1fx	Rat cerebral cortex	Raju et al. (2000)
Pro-inflammatory cytokines				
IL-1β, TNF-α, IL-16	?	? hypo versus hyper fx	Mouse colon	Barlow et al. (2016)
IL-1α, IL-1β, TNF-α	X-ray versus γ-ray (^137^Cs)	7–25 Gy/1fx	Mouse brain	Hong et al. (1995)
IL-β, TNF-α	γ-ray (^137^Cs)	10 Gy/1fx	Rat brain	Lee et al. (2010)
IL-6	γ-ray (^60^Co)	10 Gy/1fx	Human alveolar carcinoma cells (A549)	Gao et al. (2014)
IL-1β, TNF-α	α-particle (^3^He) microbeam	0.5 Gy/1fx	Human lung fibroblasts	Zhou et al. (2008)
IL-6	γ-ray (^60^Co or ^137^Cs)	9–10 Gy/1fx	Mouse plasma and lung	Van der Meeren et al. (2003)
TNF-α	γ-ray (^192^Ir)	10 Gy/1fx	Rat brain	(Kim et al. 2002)
IL-1α, TNF-α	γ-ray (^137^Cs)	0–35 Gy/1fx	Mouse brain	Moravan et al. (2011)
TNF-α	X-ray	2–20 Gy/1fx versus 10–40 Gy/5–20fx	Mouse brain	Gaber et al.(2003)
IL-1α, IL-6	X-ray	12 Gy/1fx	Mouse lung, bronchial lavage fluid, serum	Ao et al. (2009)
IL-8	α-particle (^238^Pu)	0.036–0.19 Gy/1fx	Human lung fibroblasts	Narayanan et al. (1999)
IL-1α, IL-1β, IL12p40, IL-18, TNF-α, IFN-γ	γ-ray (^60^Co)	5–20 Gy/1fx	Murine bone marrow-derived macrophages	Liu et al. (2017)
Anti-inflammatory cytokines				
TGF-β1	γ-ray (^192^Ir)	10 Gy/1fx	Rat brain	Kim et al. (2002)
IL-4, IL-5, IL-10	γ-ray (^60^Co)	5 Gy/1fx	Murine splenocytes	Han et al. (2006)
Haematopoietic				
EPO, TPO	γ-ray (^60^Co)	7.5 Gy/1fx	Mouse plasma and kidney	Barshishat-Kupper et al. (2011)
G-CSF	X-ray	12 Gy/1fx	Mouse lung, bronchial lavage fluid, serum	Ao et al. (2009)
EPO	γ-ray (^137^Cs)	4 Gy/1fx	Mouse plasma	Peslak et al. (2012)
CSF1, IL-34	X-ray	12 Gy/1fx	Patient-derived xenografts (U251, GBM12)	Stafford et al. (2016)
Prostanoids				
PGE_2_ and TXA_2_	γ-ray (^137^Cs)	35 Gy/1fx	Mouse brain	Moore et al. (2004)
PTGES	γ-ray (^137^Cs)	0–35 Gy/1fx	Mouse brain	Moravan et al. (2011)
Growth factors				
EGFR	γ-ray (^137^Cs)	2 Gy/1fx	Human breast (MDA-MB-231) and squamous carcinoma cells (A431)	Dent et al. (2003)
EGFR	γ-ray (^60^Co)	10 Gy/1fx	Human alveolar carcinoma cells (A549)	Gao et al. (2014)
Basic FGF	γ-ray (^137^Cs)	10 Gy/1fx	Rat cerebral cortex	Raju et al. (2000)
VEGF	X-ray	6 Gy/3fx	Rat cerebral cortex	Jin et al. (2014)
G-CSF	X-ray	12 Gy/1fx	Mouse lung, bronchial lavage fluid, serum	Ao et al. (2009)
Chemokines				
CXCL10	?	? hypo versus hyper fx	Mouse colon cancer cells (Colon 38)	Barlow et al. (2016)
CCL2	γ-ray (^137^Cs)	10 Gy/1fx	Rat brain	Lee et al. (2010)
CXCL1	γ-ray (^60^Co or ^137^Cs)	9–10 Gy/1fx	Mouse plasma and lung	Van der Meeren et al. (2003)
CCL2	γ-ray (^137^Cs)	0–35 Gy/1fx	Mouse brain	Moravan et al. (2011)
CXCL1, CCL2, CXCL10	X-ray	12 Gy/1fx	Mouse lung, bronchial lavage fluid, serum	Ao et al. (2009)
CCL2	γ-ray (^60^Co)	5–20 Gy/1fx	Murine bone marrow-derived macrophages	Liu et al. (2017)
Adhesion molecules				
ICAM-1	X-ray versus γ-ray (^137^Cs)	2–25 Gy/1fx	Mouse brain	Hong et al. (1995)
P-selectin, PECAM-1	γ-ray (^60^Co or ^137^Cs)	9–10 Gy/1fx	Mouse lung endothelial cells	Van der Meeren et al. (2003)
ICAM-1	γ-ray (^137^Cs)	0–35 Gy/1fx	Mouse brain	Moravan et al. (2011)
ICAM-1	X-ray	2–20 Gy/1fx versus 10–40 Gy/5–20fx	Mouse brain	Gaber et al. (2003)
P-selectin, VCAM-1	γ-ray (^137^Cs)	9 Gy/1fx	Mouse bone marrow	Mazo et al. (2002)
Enzymes				
COX-2, iNOS	α-particle (^3^He) microbeam	0.5 Gy/1fx	Human skin and lung fibroblasts	Zhou et al. (2008)
COX-2	X-ray	15 Gy/1fx	Murine microglial cells (BV-2)	Hwang et al. (2006)
COX-2	γ-ray (^137^Cs)	35 Gy/1fx	Mouse brain	Moore et al. (2004)
COX-1, COX-2, HO-1, caspase-1, GSTP-1, SOD2	γ-ray (^137^Cs)	0–35 Gy/1fx	Mouse Brain	Moravan et al. (2011)
SOD2, GST, GPX and catalase	γ-ray (^137^Cs)	0.02 Gy/fx	Human lymphoblastoid cells (AHH-1)	Bravard et al. (1999)
HO-1,GSTA-2	γ-ray (^137^Cs)	2.5–20 Gy/5fx	Murine embryo fibroblasts, fibroblast cells (NIH-3T3) and dendritic cells (DC2.4)	McDonald et al. (2010)
HO-1	γ-ray (^137^Cs)	0.1–2.5 Gy/1fx	Mouse macrophages cells (RAW264.7)	Tsukimoto et al. (2010)
Angiogenic/vascular				
VEGF	X-ray	6 Gy/3fx	Mouse brain	Jin et al. (2014)
HIF-1α and HIF-2α	γ-ray (^60^Co)	7.5 Gy/1fx	Mouse plasma and kidney	Barshishat-Kupper et al. (2011)
Basic FGF	γ-ray (^137^Cs)	10 Gy/1fx	Rat cerebral cortex	Raju et al. (2000)
Pro-fibrotic				
TGF-β1	γ-ray (^192^Ir)	10 Gy/1fx	Rat brain	Kim et al. (2002)
TGF-β1	Proton (^3^He) microbeam	0.016–0.16 Gy/1fx	Human glioblastoma cells (T98G)	Shao et al. (2008)
Acute phase proteins				
Alpha 1*-*antichymotrypsin	X-ray versus γ-ray (^137^Cs)	25 Gy/1fx	Mouse brain	Hong et al. (1995)
Hypoxia-sensing proteins				
HIF-1α and HIF-2α	γ-ray (^60^Co)	7.5 Gy/1fx	Mouse plasma and kidney	Barshishat-Kupper et al. (2011)
Coagulation				
Factors II, V, VII, VIII, IX, X, XI, XII	Proton	0–2 Gy/1fx high- versus low-dose rate	Ferret plasma	Krigsfeld et al. (2012)
TF, endothelial microparticles	X-ray	20 Gy/1fx	Human PBMC	Goldin-Lang et al. (2007)
TF, TM	γ-ray (^60^Co or ^137^Cs)	9–10 Gy/1fx	Mouse plasma and lung endothelial cells	Van der Meeren et al. (2003)
TM, PAR-1	X-ray	33.6 Gy/8fx or 67.2/16fx	Rat intestinal vascular and smooth muscle cells	Wang et al. (2002)

*AP* activator protein, *COX* cyclo-oxygenase, *CREB cyclic* adenosine monophosphate responsive element binding protein, *CSF* colony stimulating factor, *EGF* epidermal growth factor, *EGR* early growth response protein, *EPO* erythropoietin, *FGF* fibroblast growth factor, *G-CSF* granulocyte colony stimulating factor, *GM-CSF* granulocyte/macrophage colony stimulating factor, *GPX* glutathione peroxidase, *GST* glutathione S-transferase, *HIF* hypoxia-inducible factor, *HO* heme oxygenase, *ICAM* intercellular cell adhesion molecule, *NF-κB* nuclear factor-κB, *PECAM* platelet endothelial cell adhesion molecule, *PG* prostaglandin, *PI3K* phosphoinositide 3-kinase, *SOD* superoxide dismutase, *SP* specificity protein, *STAT* signal transducer and activator of *transcription, TPO* thyroid peroxidase, *TX* thromboxane, *VCAM* vascular cell adhesion molecule, *VEGF* vascular endothelial growth factor

Radiation dose, DR and fractionation modulates the aforementioned molecules. Pro-inflammatory cytokine induction requires 7–10 Gy (Hong et al. 1995), as does NF-κB (Rho et al. 2005), while up-regulation of ICAM-1 was observed with doses as low as 2 Gy (Hong et al. 1995). Fractionation of radiation regimens can influence the persistence of pro-inflammatory cytokines in the tumour microenvironment. Irradiation of C3H/HeJ and C57BL/6J murine lung with single (20 Gy) and fractionated doses (4 Gy/day) of radiation increased IL-1β within 1 h (single dose) and within 6 h (after each fractionated dose) (Hong et al. 1999). Fractionated radiation maintained a sustained up-regulation of cytokine gene expression for a longer period (Hong et al. 1999). The essential roles of ICAM-1, IL-1β, and NF-κB in the induction of the radiation-induced immune response are depicted in Fig. 2.

Radiation-induced coagulopathy including haemorrhage and microvascular thrombosis are reported in preclinical animal models, cancer patients, and Hiroshima and Nagasaki atomic bomb victims (Gutin et al. 2009; Krigsfeld and Kennedy 2013; Lai et al. 2008; Robins et al. 2006; Stupp et al. 2005). Exposure to 1.8–80 Gy ^137^Cs γ-radiation caused oxidation of Met388 at the thrombin-binding site of TM, leading to radiation dose-dependent impairment of the TM-thrombin complex formation, and insufficient activation of protein C (Ross et al. 2008). Irradiation leads to changes in levels of coagulation factors (TM, factors II, V, VII, VIII, IX, X, XI and XII) and soluble fibrin, increasing blood clotting times, haemorrhage, and microvascular fibrin clots in lung, liver and kidneys of irradiated animals (Krigsfeld and Kennedy 2013; Krigsfeld et al. 2012, 2013). Disruption of the coagulation and fibrinolytic pathways is differentially affected by radiation DR. High DR (0.5 Gy/min) increased prothrombin time (PT) due to Factor VII. In contrast, low DR (0.0083 Gy/min) increased PT values due to Factors II (prothrombin), V, and VII, and increased activated partial thromboplastin time (aPTT) mediated by Factor IX (Krigsfeld et al. 2012). As a part of the innate immune response, radiation-induced coagulation drives the immune response through the activation of caspases. Caspases cleave pro-cytokines and pro-apoptotic molecules (e.g. pro-IL-1β and Bid) activating cell death pathways and eliciting a wide-ranging immune response (Fig. 2).

### Release of tumour antigen, DAMPs and neo-antigen generation

Irradiation promotes the release of endogenous ligands, called DAMPs. These ‘danger’ signals represent a wide range of molecules including adenosine, ATP, DNA, mitochondrial DNA, receptor for advanced glycation end product ligands (e.g. heat shock proteins, S100 proteins, defensins, high-mobility group box-1 [HMGB1]), hyaluronan, oxidised phospholipids and oxidised cholesteryl esters, extracellular matrix proteins (e.g. fibronectin, hyaluronan, uric acid, and surfactant proteins) and polymorphonuclear neutrophil (PMN)-derived alarmins (Rosin and Okusa 2011; Schaue et al. 2012a). During inflammation, release of DAMPs into the extracellular space can occur via (i) expression on apoptotic cells or leakage from necrotic cells, (ii) increased synthesis and post-translational modification, and (iii) degradation of inactive precursors into toll-like receptor-mimetic degradation products (Mencin et al. 2009). Upon engagement with their receptor, DAMPs initiate self-amplified cytokine cascades leading to the activation of immunogenic cell death mechanisms (Sridharan and Schoenfeld 2015), innate immune responses (Miller et al. 2011), and inflammatory tissue (Schaue and McBride 2010).

Radiation can alter the immunogenicity of tumours and increase their susceptibility to immune recognition and clearance. Upon irradiation, tumour cells and tumour-associated endothelial cells express a wide range of ‘novel’ antigens (called ‘neoantigens’) including immune regulatory molecules—major histocompatibility complex (MHC) class I, CD80, CD20, CD94; death receptors and apoptosis-associated molecules—Fas, FasL, TRAIL, phosphatidylserine; cell adhesion molecules—integrins (α_2b_β_3,_ α_5_β_1_), cadherins (E-cadherin and α-catenin), P-selectin, E-selectin, ICAM-1, and VCAM-1; ER molecules—BiP, calreticulin as reviewed by Corso et al. (2011). While most of the studies identified were in vitro and in vivo based, few human studies are available which show similar patterns. Irradiated oral mucosa of head and neck cancer patients (60 Gy/30fx) showed increased β2 integrin, ICAM-1 and E-selectin, but not β1 integrin or VCAM-1 (Handschel et al. 1999). 100 Gy ^137^Cs γ-irradiation (DR 2 Gy/min) of freshly isolated ovarian tumours increased expression of MHCI and ICAM-1 (Santin et al. 1996). This effect could be further enhanced by priming with cytokines (TNF-α and IFN-γ) before irradiation. Notably though, the level of up-regulation of neo-antigens was different between freshly isolated ovarian tumours and continuous ovarian cell lines, with a more than 5.5-fold greater up-regulation of MHCI in fresh ovarian tumour cells (Santin et al. 1996). This suggests that neo-antigens identified in in vitro and in vivo studies should be confirmed using human tissues before progressing into therapeutic development. Whether the above neo-antigens are also ‘revealed’ and to what level in patients who receive adjuvant immunotherapy or targeted therapy with RT remains to be delineated.

Of note, DAMPS can also negate the immunogenicity of a tumour following single high-dose IR, but not fractionated IR. Doses of 12–18 Gy to breast and colorectal cancer cell lines induced three prime repair endonuclease (TREX1), impairing type I IFN production (Vanpouille-Box et al. 2017). The downstream effect is suppression of the adaptive immune response and a failure to eliminate the cancer cells. Therein TREX1 has arisen as a potential therapeutic target to enhance the RT-induced immune response to cancer.

### Inflammasomes

NLRP3 and AIM2 inflammasomes contribute to the network of DAMPs, ROS/RNS, ER stress pathways and cytokines activated by IR (Fig. 2). Activation of NLRP3 or AIM2 inflammasomes by cytoplasmic double-stranded DNA induces the release of pro-inflammatory cytokines and activates apoptosis or pyroptosis cell death. Pyroptosis is a pro-inflammatory and lytic form of cell death distinct from apoptosis in that it is dependent on caspase-1 (Bergsbaken et al. 2009).

0.5–4 Gy ^137^Cs total body γ-irradiation induces a dose-dependent increase in inflammasome activation in Mɸ, dendritic cells (DCs), natural killer (NK) cells, T-cells, and B-cells (Stoecklein et al. 2015). Following radiation, NLPR3/caspase-1 is activated from day 1 and sustained until 7-day post-radiation (Stoecklein et al. 2015). 10–20 Gy ^60^Co γ-irradiated primary cultured bone marrow-derived Mɸ showed an increased proportion of pyroptosis, elevated NLRP3 protein expression and caspase-1 activation, and significantly increased the production of IL-1*β*, IL-18, TNF-*α*, IFN-*γ*, IL-1*α*, IL-12p40 and CCL2 (Liu et al. 2017). 9.5 Gy ^60^Co γ-irradiation of C57Bl/6 mice induced caspase-1 cleavage in spleen marginal zone cells (rich in Mɸ and follicular DCs), IL-1β, and induced death in the mice (Liu et al. 2017). Akin to the NLRP3 inflammasome, radiation induces activation of the AIM2 inflammasome and caspase-1 mediated pyroptosis (Fernandes-Alnemri et al. 2009; Lamkanfi and Dixit 2014). AIM2/ASC/caspase-1 mediates intestinal epithelial cells and bone marrow cell death from 14.2 Gy subtotal body irradiation (Hu et al. 2016). These data show that radiation-induced activation of either the NLRP3 or AIM2 inflammasomes will trigger immunogenic cell death mechanisms apoptosis and pyroptosis.

### Bystander and abscopal effect

The interconnected inflammatory pathways elicited by irradiation of tumour tissue are summarised in Fig. 2. Ultimately, the tumour response to IR is dictated by the adjacent cells in a multi-cellular response (‘bystander effect’) and systemically at sites distant to the site of irradiation (‘abscopal effect’). Evidence of the bystander effect began with the observation that 0.31 mGy ^238^Pl α-particle irradiation of Chinese hamster ovary cells induced sister chromatid exchanges in 30% of cells, yet only 1% of cells were irradiated (Nagasawa and Little 1992). In X-ray irradiation, a dose of 2 Gy elicited a biologically similar effect. This has since been repeated by other laboratories with different radiation sources including heavy ion radiation. ^3^He single-cell microbeam irradiation of human T98G glioblastoma cells leads to 40% of cells demonstrating increased NO levels, whilst only 1% of cell nuclei were targeted (Shao et al. 2003).

#### Bystander effect

Intracellular communication via gap junctions, and intercellular signalling by means of a range of cell-derived soluble factors have been implicated in modulating the bystander effect. These include ROS NO^·^, O_2_^·−^, H_2_O_2_ (Burdak-Rothkamm et al. 2007; He et al. 2012; Narayanan et al. 1997; Shao et al. 2003, 2007, 2008), [Ca^2+^]_f_ (Shao et al. 2006); second messenger cyclic adenosine monophosphate (He et al. 2014); cytokines IL-1α, IL-6, IL-8, TGF-β1, TNF-α (Burdak-Rothkamm et al. 2007; Dong et al. 2015; Ivanov and Hei 2014a; Narayanan et al. 1999; Shao et al. 2007, 2008; Shareef et al. 2007); prostanoid PGE_2_ (Zhou et al. 2008); transcription factor NF-κB (Fu et al. 2016; Zhou et al. 2008); enzyme cyclo-oxygenase-2 (COX-2) (Zhou et al. 2005, 2008); tyrosine kinase MAPK and PKC (Baskar et al. 2008; Fu et al. 2016; Zhou et al. 2005); tumour suppressor p53 (He et al. 2011); DNA damage repair ataxia telangiectasia mutated protein (Burdak-Rothkamm et al. 2008; Hagelstrom et al. 2008), ataxia telangiectasia and Rad3-related protein (Burdak-Rothkamm et al. 2007), DNA-dependent protein kinase (Hagelstrom et al. 2008); death receptor ligand soluble TRAIL (Ivanov and Hei 2014a, b; Shareef et al. 2007); and cytochrome c (He et al. 2011, 2012). These modulators initiate cell death or senescence in neighbouring tumours cells. Grid therapy, minibeam/microbeam RT and spatial modulation (delivery of radiation to a treatment area that is divided into several smaller fields) with sharp dose gradients may enable greater tumour cytotoxicity and reduce radiation-induced injury to surrounding healthy tissues by enhancing the bystander effect whilst preserving endothelial cell and surrounding healthy tissue function (Asur et al. 2015; Mackonis et al. 2007; Trainor et al. 2012; Zhang et al. 2014).

#### Abscopal effect

While the local bystander effect is being touted as a way of evoked greater tumouricidal activity while preserving healthy normal tissue, by way of reducing radiation-induced injury to endothelial and parenchymal cells, the systemic abscopal effect has received greater scepticism with primarily anecdotal case study reports and few preclinical studies. Mole first described the abscopal effect as a radiation-induced tumour effect “at a distance from the irradiated volume but within the same organism” (Mole 1953). A recent meta-analysis study of the preclinical data available to date conclude that the abscopal effect is linked to the biological effective dose of radiation, whereby a dose of 60 Gy has a probability of 50% for observing the abscopal effect (Marconi et al. 2017). Perhaps the greatest body of support for the abscopal effect comes from a 2015 proof-of-principle clinical trial. Patients with solid metastatic cancer received 35 Gy/10fx X-ray radiation to one metastatic lesion and adjuvant GM-CSF (125 µg/m^2^), before repeating the regimen on a second metastatic lesion. Of the 41 patients enrolled, 11 patients achieved abscopal response denoted as a 30% reduction in the maximal tumour diameter in the best responding lesion (Golden et al. 2015).

Both the abovementioned preclinical and clinical studies involved a range of tumour types breast, colon, lung, fibrosarcoma, pancreas, melanoma, head and neck cancer (Marconi et al. 2017), non-small cell lung, breast and thymic (Golden et al. 2015) indicating that the abscopal effect is plausible and can be elicited in many cancer types. A similar meta-analysis encompassing case reports, a retrospective study and preclinical data surmised that issues concerning radiation and immunotherapy dosage, timing, patient selection and toxicity need to be resolved before the abscopal effect can become clinically relevant (Reynders et al. 2015).

The abscopal effect is mediated by antigen presenting cells DCs, Mɸ and CTLs activated by inflammatory mediators (e.g. cytokines, DAMPs, ROS/RNS) from the irradiated tumour microenvironment and travelling to tumour-draining lymph nodes and distant non-irradiated sites. In the lymph nodes, DCs and Mɸ activate effector T-cell populations through MHC presentation and secretion of the soluble tumour modulating molecules described for the bystander effect above. Following activation and expansion in the lymph nodes CTL, NK cells and Th-cell populations travel to distant tumour sites and elicit a pro-inflammatory response leading to reductions in tumour growth at non-irradiated sites (Demaria and Formenti 2016; Grass et al. 2016; Hu et al. 2017; Ng and Dai 2016; Siva et al. 2015). This effect is enhanced by PDL-1/PD-1 immunotherapy which blocks the immunosuppressive signal from the tumour cells, enabling a persistent pro-inflammatory and tumourcidal microenvironment (Park et al. 2015). A summary of pre-clinical studies demonstrating the abscopal effect following combined RT and immunotherapy can be found in a recent review by Ngwa et al. (2018).

## Inflammatory and immune system cellular response

**Fig. 3:**
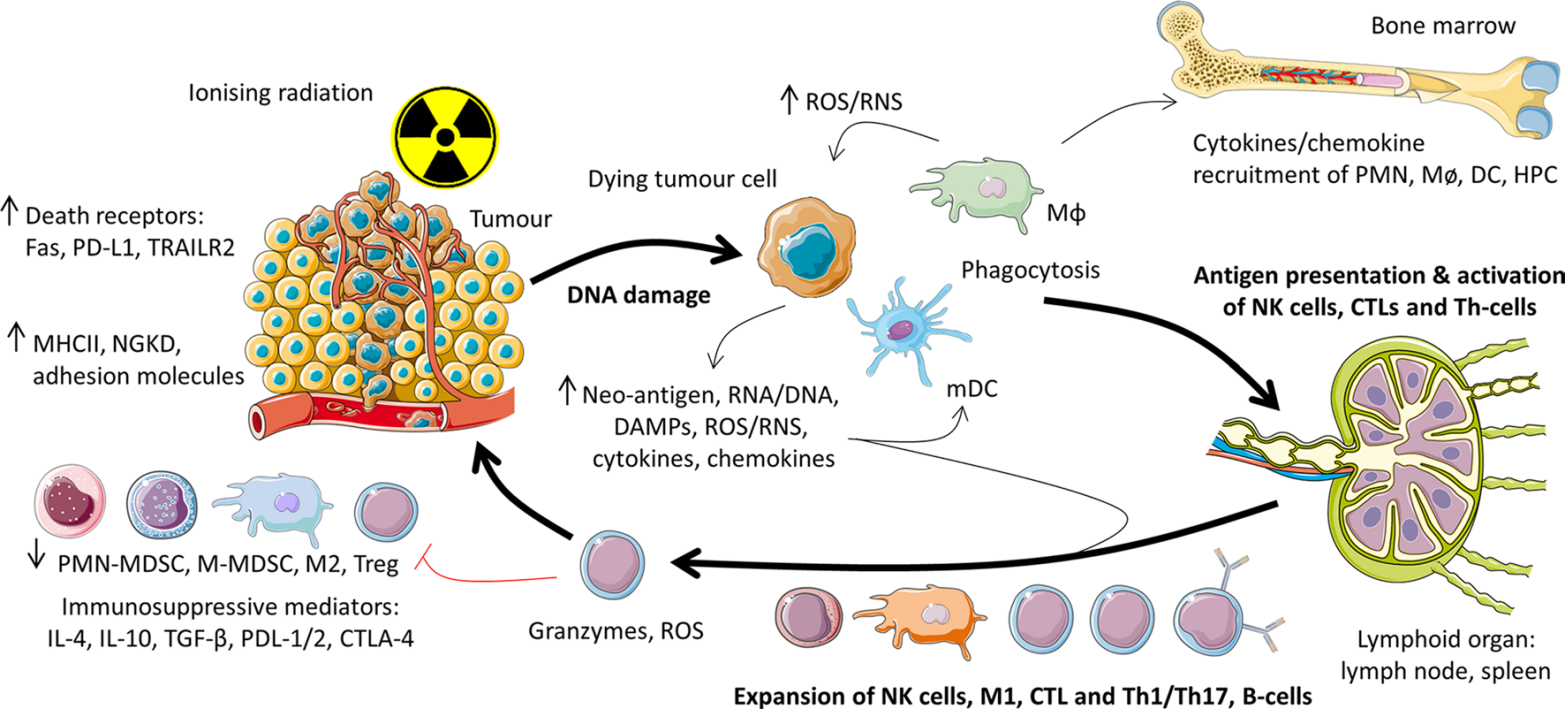
Role of the innate and adaptive immune cells following the irradiation of tumour cells. *HPC* haematopoietic progenitor cells. Bold text and arrows denote the essential pathway of RT-induced immune activation by (i) direct or indirect (ROS-RNS-mediated) DNA damage, (ii) antigen presentation and activation by DCs, and (iii) expansion and specificity of the adaptive immune response. Image modified from (Kamrava et al. 2009) and prepared using Servier Medical Art (https://smart.servier.com/)

To date this review has covered inflammatory mediators and cell death mechanisms initiated and elicited by IR. While immune cell populations also contribute ROS/RNS, DAMPS, cytokines/chemokines and inflammasome activation in the irradiated tumour microenvironment (Fig. 3), this section focusses on the effect of IR on innate and adaptive immune cell recruitment, expansion, polarisation/differentiation, and functions not already addressed elsewhere in the review.

### Innate immune cells

IR actives resident Mɸ (including microglia) via ROS/RNS, DAMPS, cytokines/chemokines and stimulates the release of haematopoietic progenitors from the bone marrow and the infiltration of PMN, Mø and DCs in the tumour microenvironment (Fig. 3). Low-dose total body X-ray irradiation (0.075 Gy) induces the recruitment of haematopoietic progenitor cells from the bone marrow into peripheral blood at 48–72 h of post-radiation. This is mediated by the release of G-CSF and GM-CSF (Li et al. 2004). Following 4 Gy X-ray irradiation, there was transient production of the PMN chemoattractants CXCL2 and CXCL8, and the infiltration of PMNs (Uchimura et al. 2000). Mɸ are recruited into irradiated tumour tissue at both acute and late stages through Mø/Mɸ chemoattractants CCL2, CCL5, CCL7, CCL8, CXCL12, VEGF and CSF-1 (Kioi et al. 2010; Kozin et al. 2010; Sica et al. 2008; Xu et al. 2013). As for PMN and Mø, immature DCs are recruited by a range of chemokines generated during pro-inflammatory response in the irradiated tumour microenvironment including CCL3, CCL4, CCL7, CCL13, CCL15, CCL20, CCL5, CCLZS and CXCL12 (Caux et al. 2000; Kioi et al. 2010; Kozin et al. 2010; Vicari et al. 2004).

Radiation polarises Mɸ to pro-inflammatory Mɸ type 1 (M1) and immunosuppressive Mɸ type 2 (M2) phenotypes. Following radiation, Mɸ activate p53-dependent ROS/RNS pathways (Lorimore et al. 2001), and the COX-2 pathway mediated by TNF-α and FasL (Rastogi et al. 2012b). Tumour-associated Mɸ (TAMs) in irradiated tissue polarise to a pro-inflammatory M1 phenotype (iNOS^+^), produce ROS/RNS and the secretion of pro-inflammatory cytokines IL-6, IL-12, TNF-α and IFN-γ (Farooque et al. 2016; Klug et al. 2013; Rastogi et al. 2013; Tsai et al. 2007). Yet, tumour cells and the irradiated tumour microenvironment also produce M2 activators (IL-4, IL-10, IL-13, TGF-β, and PGE_2_). TAMs in the irradiated tumour microenvironment express higher levels of M2 markers (Arg-1, COX-2), and promote early tumour growth in vivo (Tsai et al. 2007). TAMs phagocytose radiation-induced apoptotic cells and debris. This leads to their activation, marked by membrane ruffing, increased lysosome number and size, increased Mɸ lysosomal acid β-galactosidase activity, and iNOS/ROS/RNS activity (Lorimore et al. 2001). Radiation-induced apoptosis of murine L1210 leukaemic cells or human Jurkat T-cells increased the engulfment/phagocytosis by M1s ~ fourfold (and to a lesser extent M2) (Rastogi et al. 2013). Engulfment of apoptotic cells further polarises the M1 versus M2 phenotypes.

Fractionation of radiation changes the timeline for M1 and M2 involvement. Following a 25 Gy single fraction X-ray dose (DR 2–3 Gy/min) in murine TRAMP-C1 prostate cells, M2 markers (Arg-I and COX-2) increased transiently at 24 h before a larger persistent infiltration developed from day 3 (Tsai et al. 2007). M1 marker (iNOS) increased from day 3 and progressively increased up to 3-week post-irradiation. In contrast, fractionated radiation of 60 Gy/15fx saw a similar increase in M2 up to 5-day post-irradiation, but M1 presence was delayed—appearing after ten fractions (2 weeks post-irradiation) (Tsai et al. 2007). Some care needs to be taken when translating murine model to human studies. In addition to strain differences in radiosensitivity, oxidative stress response, DNA repair, apoptosis and cytokine production, differences in immune regulation are also noted (Ao et al. 2009; Haston et al. 2005). In vivo, murine 4 Gy γ-radiation exposure is associated with genotype-dependent bone marrow-derived Mɸ activation. Radiation further induced a CBA/Ca M1 phenotype (increased iNOS, IL-6, TNF-α, ROS/RNS) and C57Bl/6 M2 phenotype (reduced iNOS, increased arginase and TGF-β) (Coates et al. 2008; Rastogi et al. 2012a). In patients, 72-86Gy irradiation of extratumoural ocular tissue or gliomas with ^60^Co, ^106^Ru or ^125^I heavy ions increased CD68^+^ Mɸ infiltrates, however M1/M2 subtype was not delineated (Julow et al. 2007; Toivonen and Kivela 2012).

Akin to Mɸ, DCs represent a spectrum of cell phenotypes which are involved in the tumour immune response and altered by IR. These include myeloid DCs, plasmacytoid DCs, monocytic myeloid-derived suppressor cells (M-MDSC) and PMN-MDSCs. 20 Gy/1fx irradiation of B16 melanoma tumour-bearing mice determined that radiation increased CD11c^+^ mDC migration to tumour-draining lymph nodes, and promoted their maturation (up-regulation of MHCII) (Lee et al. 2009). No effect was seen on plasmacytoid DCs. While the cell-specific markers of MDSCs are still being elucidated (Bronte et al. 2016), this heterogeneous class of DCs provides immune suppression of Th-cells and CTLs in the tumour microenvironment (Movahedi et al. 2008; Solito et al. 2012). Radiation-induced cell death externalises DAMPs such as HMGB1, ATP and calreticulin on the tumour cells surface and the release of ROS/RNS (Fig. 3) (Frey et al. 2014; Inoue and Tani 2014; Lotze et al. 2007). These promote DC maturation and the activation of effector T-cell responses via DC-derived co-stimulatory molecules and cytokines (Tan et al. 2013). Radiation-induced ROS/RNS enhance the immunosuppressive activity of MDSC via up-regulation of the Keap1/nuclear factor erythroid—2-related factor-2 sensor of oxidative stress (Beury et al. 2016). Following irradiation, murine 1D8 ovarian carcinoma cells up-regulate the activation marker CD80 and suppress CD4^+^CD25^+^ regulatory T-cells (Tregs) via engagement of CD152 (CTLA-4) (Yang et al. 2006). M-MDSC immunosuppression of T-cell responses is dependent on M-MDSC STAT1 and the production of IFN-γ and NO, while PMN-MDSC immunosuppressive activity requires IFN-γ (Movahedi et al. 2008). High-dose X-ray radiation (30 Gy/1fx; DR 1.21 Gy/min) of CT26 or MC38 colon tumour-bearing mice can overcome the MDSC suppressive activity and returns the CTL population required for tumour eradication (Filatenkov et al. 2015). This change was driven by CD8^+^ DCs cross-presentation and IFN-γ secretion, and CD40L expressing Th-cells. These highlight that IR is a critical activator of innate immune cells for the macrophagic removal of apoptotic cells, drive the antigen-specific response to tumour cells through DC antigen presentation, and to alleviate immunosuppressive signalling by MDSCs in the tumour microenvironment (Fig. 3).

### Adaptive immune cells

Polarisation and activation of Mɸ and DCs by DAMPs and ROS/RNS in the irradiated tumour environment are critical for driving the adaptive immune response (Frey et al. 2014; Inoue and Tani 2014; Lotze et al. 2007). Upon activation, Mɸ and DCs migrate to the tumour-draining lymph nodes and present tumour-specific antigen on MHCI or MHCII molecules to T-cells and B-cells (Fig. 3).

IR can induce the expression of neo-antigens by tumours cells which can activate NK cells directly (NKGD and MHCI), or indirectly through DAMPs and ROS/RNS released from dying tumour cells. 15 Gy X-ray irradiation of murine B16 melanoma tumour-bearing mice increased the proportion of apoptotic and necrotic tumour cells, as well as B-cells, NK cells, Th-cells and CTLs in tumour-draining lymph nodes when irradiated in conjunction with hyperthermia (41.5 °C for 1 h) (Finkel et al. 2016). In canine osteosarcoma, 10–20 Gy irradiation dose-dependently increased NK cytotoxicity (Canter et al. 2017). Low-dose RT (2 Gy) was found to prime NK cells and increased NK cell number and cytotoxicity of tumours following autologous adoptive transfer into dogs (Canter et al. 2017).

Akin to NK cells, CTLs are a critical tumourcidal cell through their expression of death receptor and the release of granules containing perforin and granzymes. 30 Gy/1fx radiation of murine colon tumours enhanced the CTL infiltrate 3.7-fold and is dependent on antigen cross-presentation by CD8^+^ DCs and IFN-γ (Filatenkov et al. 2015). Fractionated RT of 30 Gy/10fx reduced CD8^+^ T-cell infiltrates 2.4-fold compared to non-irradiated animals and increased fatality. Murine B16 melanomas irradiated with 20 Gy/1fx X-ray reduced tumour size by ~ 30% and increased survival. This effect required CTLs and the migration of CD11c^+^ DCs to draining lymph nodes to prime T-cells (Lee et al. 2009). Of note, this study went on to demonstrate that chemotherapy abolished DC-mediated priming of T-cells and ablated the RT-induced anti-metastatic effect of CTLs, and fractionated 20 Gy/4fx failed to induced tumour regression (Lee et al. 2009). The authors speculated that fractionated RT may kill off tumour infiltrating CTLs, and raised the question of the relative biological effectiveness of 20 Gy/1fx versus 20 Gy/4fx in activating the inflammatory/immune response.

Th-cells comprise pro-inflammatory (Th1 and Th17), anti-inflammatory (Th2) and immunosuppressive subsets (Treg). The former subsets play a role in tumour eradication, while Tregs support tumour growth principally through the secretion of TGF-β which suppresses pro-inflammatory and tumourcidal immune cell subsets (Chen et al. 2005). In addition, radiation increases Treg hydrolysis of the pro-inflammatory DAMP signal ATP into immunosuppressive/negative feedback signal adenosine through induced expression of cell surface ecto-nucleotidases (CD39 and CD73) (Mandapathil et al. 2009; Wennerberg et al. 2015). In general, TBI or local irradiation increases the Treg population in mice and humans (Billiard et al. 2011; Kachikwu et al. 2011; Schaue et al. 2008, 2012b; Tomura et al. 2010), though the converse is noted in some studies (Cao et al. 2009; Wennerberg et al. 2015).

In the other Th-cells populations 2.5 Gy X-ray TBI (DR 1.3 Gy/min) increased Th2 cells from day 5 and Treg and Th17 cells from day 8 post-irradiation, while Th1 cells and IFN-γ production was reduced (Zheng et al. 2015). This appears to be driven by radiation-induced alterations in transcription factor expression. 5 Gy ^60^Co irradiation up-regulated Th2 transcription factors c-MAf and GATA-3 and down-regulated mRNA interferon regulatory factor-1 and IFN-γ mRNA and Th1 transcription factors STAT1 and STAT4 phosphorylation (Han et al. 2006). Murine 18 Gy whole thorax X-ray irradiation (DR 0.54 Gy/min) leads to changes in T-cell populations at 6 h, 24 h and 7 days in lung tissue, with Th1 and Th17 populations increased in lung tissue but lower in the bronchoalveolar lavage fluid (Paun et al. 2015). Notably strain dependent differences were observed. The difference in Th-cell populations are partly indicated by the radiosensitivity of the cell subsets to radiation mediated by p53-dependent cell death (Yao et al. 2011). NK cells, naive T-cells and B-cells is highly radiosensitive, while effector T-cells and NK/T-cells, are more radioresistant (Belka et al. 1999; Kachikwu et al. 2011; Qu et al. 2010; Yao et al. 2011). Of the effector T-cell populations, Th-cells are more radioresistant than CTLs (Yao et al. 2011). However, it may equally be the CTL:Th (CD8^+^:CD4^+^) ratio and diversity of the TCR repertoire, rather than inherent differences in T cell radiosensitivity, that is critical in the elimination of cancer cells. This is highly relevant when considering which model to use for pre-clinical studies of combined RT and immunotherapy (Rudqvist et al. 2018).

B-cells are highly radiosensitive and have a diminished capacity for antigen presentation and antibody production after irradiation. Localised 26 Gy/13fx irradiation of patients with seminoma resulted in a 5.7-fold and 11-fold decrease in CD19^+^ B-cells after 7fx and 13fx, respectively (Belka et al. 1999). B-cell numbers remained 2.4-fold lower than baseline levels up to 4 months after RT. B-cells exposed to 10–33 Gy ^137^Cs γ-radiation show deficits in antigen presentation and activation of T-cells, despite displaying functional MHCII (Ashwell et al. 1988) and increased expression of the B-cell antigen (CD20) (Kunala and Macklis 2001). Occupational radiation exposure of miners to ^222^Rn reduced immunoglobulin (Ig) IgG and IgM serum levels after 1 year, but after 2 years IgG increased and exceeded the baseline levels. IgA levels did not change. The hypogammaglobulinemic period (low IgG) was followed by an increased in respiratory tract infections (Andrlikova et al. 1975). Similar changes to serum antibody levels have been observed at 5–15 years of post-irradiation (Wagner et al. 1978). Changes in Ig production are sensitive to IR and have implications for opportunistic infections as well as cancer patients with comorbid autoimmune disorders (Andrlikova et al. 1975).

## Effect of radiation delivery on the immune response

**Fig. 4:**
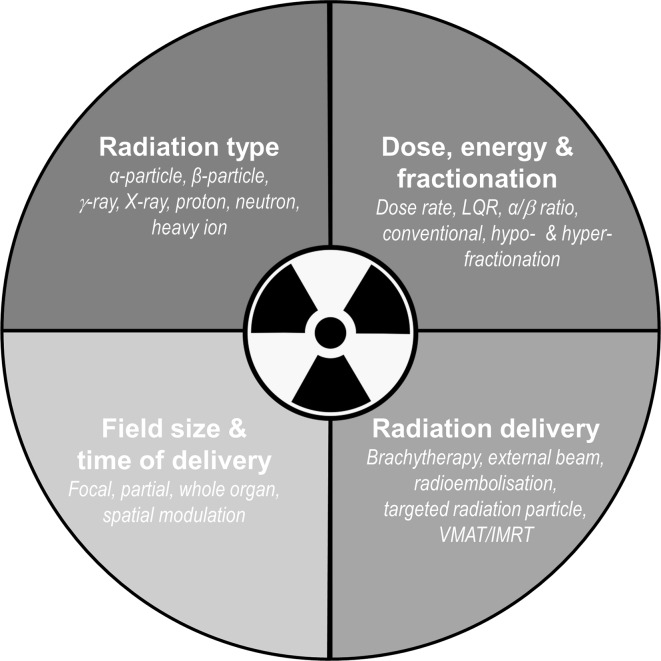
Aspects of radiation delivery that modulate the inflammatory/immune response

The effect of IR on inflammation and the immune response is dictated by the radiation type, dose, DR, intensity/fractionation, delivery method, field size, and total cumulative dose. In this review, these have been highlighted where available in the literature. Further elucidation of how the above factors alter the relative biological effectiveness of IR on different cancers is needed (Fig. 4), bearing in mind that the radiation total or cumulative dose is in itself dependent on the indication, site of tumour, cancer subclassification, tissue/organ radiosensitivity and the patient’s performance status.

While the mechanism remains to be fully elucidated Flash RT, the use of ultra-high DR, is an interesting prospect to further reduce the normal tissue toxicity and maintain tissue function (Durante et al. 2018). Thoracic IR of mice using 15 Gy conventional X-ray (DR 1.8 Gy/min) or 20 Gy Flash RT (DR > 2400 Gy/min; pulse rate < 500 ms) provided comparable lung tumour control, but Flash RT produced fewer fibrotic lesions (Favaudon et al. 2014). In a similar study, 10 Gy murine whole brain FLASH RT (DR > 6000 Gy/min; pulse rate 1 µs) preserved spatial memory and hippocampal neurogenesis when compared to conventional RT (6 Gy/min) (Montay-Gruel et al. 2017). The effect was absent if DR < 1800 Gy/min were used.

While in vitro and in vivo studies utilise the clinical radiation dose/fractionation, the biological implication of DR and differences in the radiosensitivity (α/β ratio) of the cell line used in the research, relative to the patient tumour, needs to be considered. For example, the 1.8–2 Gy per fraction regimen was based on human prostate cancer cells having a low α/β ratio. Assessment of 5,969 human primary prostate cancers shows an α = 0.009–0.061 Gy(− 1) and an α/β = 0.9 to 2.2 Gy (Miralbell et al. 2012), while prostate cancer cell lines demonstrate an α = 0.09–0.35 Gy((− 1), and the α/β = 1.09 to 6.29 Gy (Carlson et al. 2004). If not taken into consideration, the wider range of α/β ratio in cell lines may have a significant bearing on the interpretation of radiation-induced modulation of the inflammatory/immune response and relative biological effectiveness of different regimens, and ultimately translation of the data to the clinical setting.

## Concluding remarks

It is clear that IR initiates and modulates the inflammatory and immune response through a range of soluble and cell-derived factors in the tumour microenvironment. Evident from this review, greater detail in the reporting of radiation type, dose, DR and validation strategies are appearing in manuscripts. However, a couple of changes could be made to existing strategies to provide greater understanding of the inflammatory and immune response to IR, which will have an impact on patient survival in the next 5 years.

Where feasible, serial biopsies should be included in clinical trials to assess not only longitudinal tumour morphological characteristics (Ki67, vascularity, changes in subtype due to cellular changes) but also the changing immune response in the tumour microenvironment before, during and after RT with/out adjuvant therapies, to advise where therapeutic regimens could be improved. This will identify those patients who do and do not respond to a treatment regimen and guide the most appropriate selection of targeted therapies and immunotherapies in relevant patient populations.

While RT-induced immune-related adverse events are beyond the scope of this review, it should be mentioned that a body of evidence is emerging for cardiotoxicity from thoracic RT and combined RT with anti-PD-1 immunotherapy or chemotherapy regimens (Contreras et al. 2018; Du et al. 2018) and highlights the need for greater understanding of the acute and long-term adverse effects of RT/immunotherapy in both tumour and non-tumour tissue compartments. In the preclinical setting, the availability of preclinical irradiators that utilise the clinical technology and treatment planning systems, SARRP^™^ by Xstrahl LifeSciences and RAD SmART™ by Precision X-ray, will improve our understanding of RT-induced immune-related adverse events, as well as the accuracy and reproducibility of RT performed but only if radiobiology/radiooncology principles are also applied, such as the radiosensitivity α/β ratio, beam arrangement, and tumour and target volumes.

Finally, it remains to be seen whether activation of bystander effects using spatially modulated fields, and/or harnessing the abscopal effect, are viable treatment strategies. How these and different types of radiation, and combined adjuvant targeted-therapy and immunotherapy influences the inflammatory and immune response needs to be further explored and integrated into clinical practice.
